# Birth in a Health Facility –Inequalities among the Ethiopian Women: Results from Repeated National Surveys

**DOI:** 10.1371/journal.pone.0095439

**Published:** 2014-04-21

**Authors:** Elias Ali Yesuf, Mirkuzie Woldie Kerie, Ronit Calderon-Margalit

**Affiliations:** 1 Department of Health Services Management, Jimma University, Jimma, Ethiopia; 2 Braun School of Public Health and Community Medicine, Hebrew University, Jerusalem, Israel; University of Alabama at Birmingham, United States of America

## Abstract

**Background:**

Uptake of health facilities for delivery care in Ethiopia has not been examined in the light of equality. We investigated differences in institutional deliveries by urbanity, administrative region, economic status and maternal education.

**Methods:**

This study was based on nation-wide repeated surveys undertaken in the years 2000, 2005, and 2011. The surveys used a cluster sampling design. Women of reproductive age were interviewed on the place of their last delivery. Data was analyzed using logistic regressions to estimate the weighted association between birth in a health facility and study's predictors.

**Results:**

Utilization of health institutions for deliveries has improved throughout the study period, however, rates remain low (5.4%,2000 and 11.8%,2011). Compared with women from rural places, women from urban areas had independent OR of a health facility delivery of 4.9 (95% CI: 3.4, 7.0), 5.0 (95% CI: 3.6, 6.9), and 4.6 (95% CI: 3.5, 6.0) in 2000, 2005, and 2011, respectively. Women with secondary/higher education had more deliveries in a healthcare facility than women with no education, and these gaps widened over the years (OR: 35.1, 45.0 and 53.6 in 2000, 2005, and 2011, respectively). Women of the upper economic quintile had 3.0–7.2 times the odds of healthcare facility deliveries, compared with the lowest quintile, with no clear trend over the years. While Addis-Ababa and Dire Dawa remained with the highest OR for deliveries in a health facility compared with Amhara, other regions displayed shifts in their relative ranking with Oromiya, SNNPR, Afar, Harari, and Somali getting relatively worse over time.

**Conclusions:**

The disparity related to urbanity or education in the use of health facility for birth in Ethiopia is staggering. There is a small inequality between most regions except Addis Ababa/Dire Dawa and sign of abating inequity between economic strata except for the richest households.

## Introduction

Giving birth in a health facility has been suggested as the single most effective intervention that may reduce maternal mortality below 200 per 100 000 live births [1]. An analysis of Afghan data showed that the combination of birth in a health facility together with improved transportation and increased modern coverage of contraceptive services could prevent 3 out of 4 maternal deaths [2].

Ethiopia has expanded the physical accessibility of maternal healthcare services. The number of health centers, which are part of primary healthcare units, has increased substantially in the country from 412 in 1997 to 2689 in 2010 [3]. Nevertheless, maternal mortality has decreased from 871 per 100, 000 live births in 2000 [4] to only 676 in 2011 [5], and thus Ethiopia has not joined a host of countries on-track to achieving the maternal mortality targets of the 5^th^ Millennium Development Goal [6].

Ethiopia has set a national target of reaching an institutional delivery rate of 32% by 2010 [7], however, according to recent studies, only about 12% of births were reported to take place in health facilities in north-western and south-eastern Ethiopia [8], [9]. Both studies have reported inequalities in the use of a health facility for birth based on area of residence, antenatal care (ANC) visits and maternal education [8], [9]. Other studies from elsewhere in Ethiopia have reported inequalities based on area of residence and maternal education, reporting that women from rural areas and lower levels of education being more likely to give birth at home than women of urban residence or higher level of education [10], [11]. These studies, however, were small sized, conducted in single communities [8]–[11], precluding generalizability to the whole country. Moreover, comprehensive data demonstrating time trends of inequalities on birth in health facilities is lacking.

We therefore aimed to study the inequalities in the use of a health facility to give birth in Ethiopia by place of residence, administrative region, household economic status, and maternal education. We have further evaluated whether the inequalities (if any) were widening over time.

## Materials and Methods

### Ethics Statement

This study is based on nation-wide data collected by the Central Statistical Agency (CSA) of Ethiopia and Inner City Fund (ICF) International of the United States of America in the years 2000, 2005, and 2011 in Ethiopia. The data is available as a public repository (data is available online at: http://www.measuredhs.com/data/available-datasets.cfm). The surveys on which this study was based were approved by the Institutional Review Boards of the Ethiopian Health and Nutrition Research Institute (EHNRI), Ministry of Science and Technology of Ethiopia, and ICF International. During the surveys participants were informed about the purpose and duration of the interviews. They were also told that participation was voluntary, and that they could withdraw from the study at any time if they wished to do so. Furthermore, participants were notified that the information provided in the interviews was to be de-identified, to keep their anonymity. They were asked whether they understood the nature of the survey and any questions thereof were explained to them. After these processes the interviewer asked them to consent to participate. For those who consented to participate, the interviewer signed and dated the consent form and proceeded with the interview.

### Design and Setting

We analyzed data from nation-wide repeated cross-sectional surveys, the Ethiopia Demographic and Health Surveys (EDHS), which were undertaken by the Inner City Fund (ICF) International, Calverton, United States of America, and the Central Statistical Authority (CSA) of Ethiopia. Data was collected in February-June 2000, April-August 2005, and December 2010–June 2011 (data is available online at: http://www.measuredhs.com/data/available-datasets.cfm) [12].

Data was collected in a community setting and included all administrative regions, as well as both urban and rural areas. Women of reproductive age living in any part of Ethiopia who have had at least 1 live birth in the five years prior to the point of time of data collection were eligible to participate in the surveys.

The participants were selected by a stratified-cluster sampling design. The sampling was a two stage process. The sampling frames for the first stage of both the 2000 and the 2005 surveys were the Census Enumeration Areas (CEAs) of the 1994 Population and Housing Census [13]. The sampling frame for the first stage of the 2011 survey was based on the CEAs of the 2007 Population and Housing Census of Ethiopia [14]. For all the surveys, CEAs were selected by systematic random sampling, followed by, a systematic random sampling to select households in these CEAs. Details on the design-, frames-, and process of sampling-of each survey are described elsewhere [15].

Participants were interviewed in-person using structured questionnaires that included information regarding the place of birth of their last live birth. Response rates for the three surveys ranged from 95% in 2011 to 97.8% in 2000. The response rates did not vary by area of residence or administrative region.

### Study Outcome

The outcome of this study was place of birth (home versus health facility). Health facility birth was defined as using a hospital, health center, health post, or a clinic for giving birth to the last live birth in the five years preceding the survey as stated by the participant.

### Study Predictors and Confounders

We have selected predictors that are routinely used to monitor progress on coverage and inequities in coverage of countries towards the provision of maternal, newborn, and child health care by Countdown to 2015 report [16].

The predictor variables in this study were place of residence [urban versus rural]; administrative region [Tigray, Afar, Amhara, Oromia, Somali, Benshangul Gumuz, Gambela, Southern Nations, Nationalities and Peoples Region (SNNPR), Harari, and city administrations (Addis Ababa and Dire Dawa)]; and education of the participant. The economic status of the household in which the participant spent the previous night was also a predictor.

For the purpose of this study, we have grouped the administrative regions based on culture, geography, and urbanity. Afar, Somali, and Harari were grouped together because they have a largely Muslim population and are located in eastern Ethiopia. Moreover, Gambela and Benshangul-Gumuz are located in Western Ethiopia and thus were grouped together. Administrative regions with a predominantly urban population, such as Addis Ababa and Diredawa were grouped together.

As suggested by Filmer and Prichet, household economic status was measured as an index based on household assets (television, radio); type of cooking fuel; type of toilet facility; utilities (source of water supply, electricity); and the roof- and floor-materials of the house [17]. Household economic status is presented in five quintiles (poorest, poorer, middle, richer, and richest). Education of the mother (no education, primary, secondary/higher) was measured based on the highest level of formal schooling achieved.

We have identified confounding variables from the literature. The main confounding variables were age in years (15–24, 25–34, 35+), birth order (1, 2–4, 5+), ANC status (no ANC, at least one ANC without complication counseling, and at least one ANC with complication counseling). Furthermore, we have controlled for economic autonomy and attitude towards spousal violence as potential confounders.

We have used employment as an indirect measure of one dimension of women’s autonomy in household decisions because employed women paid in the form of cash have a higher say in household decision in low-income countries such as, Nepal and Bangladesh [18]. A composite variable was constructed comprising employment status and working conditions (type of employer, location of work, contract type and payment method). Eventually, a total economic autonomy score was generated by summing up the individual scores of employment status and components of the working conditions. Economic autonomy is presented as no autonomy, low autonomy, and high autonomy.

Finally, attitude towards spousal violence (yes versus no) was formulated as a composite variable based on the respondent’s attitude on a range of issues related with violence involving beatings.

More information on how the household economic status, economic autonomy, and attitude towards spousal violence were formulated can be found elsewhere.

### Statistical Analysis

The statistical analyses were done taking into account the cluster sampling design. All the analyses were scaled for individual sample weights (inverse of the probability of selection of household*inverse of response rate of the household response rate group*inverse of response rate of individual response rate group).

Weighted rates of birth in a health facility were calculated and stratified by study predictors and confounders. Binary logistic regressions were used to calculate crude odds ratios (OR) for birth in a health facility and 95% Confidence Intervals (CIs). Adjusted ORs for birth in a health facility were determined using Generalized Linear Models which included binary logistic models scaled for sampling weights. All statistical analyses were performed using SPSS software v.16 (IBM Corporation, Chicago, IL). A two sided p value<0.05 was considered statistically significant.

### Approach to Modeling

The modeling sought to determine the crude effects of the predictors. It also sought to measure the main-effects of predictors to account for relations between them. In coming up with the independent effects of the predictors, confounders were accounted for in the final model.

Model 1 estimates the crude effects of each of the predictors. Model 2 predicts the main effects of the predictors without accounting for the confounders. Model 3 shows the independent effects of the predictors by controlling the confounders.

## Results

### Description of the Participants

A total of 7245 (weighted: 7978), 6589 (weighted: 7307) and 7764 (weighted: 7908) women of reproductive age were interviewed about their place of birth for the last live birth in the five years preceding 2000, 2005 and 2011, respectively.

In the 2000 survey, the mean age of the respondents was 29.7(standard deviation = 7.5) years. Three administrative regions (Oromia, Amhara and SNNPR) have accounted for 88% of the participants. About 89% of participants were from rural areas and 82% had no education. Ethiopian Orthodox Christians made up for about half of the participants. Finally, about 90% of the respondents were married and about 42% had parity of 5 or more children. Similar socio-demographic patterns were observed in the 2005 and 2011 surveys (data not shown).

### Rates- and Inequities-of Birth in Health Facilities

According to the 2000 survey, only 5.4% (431 out of 7978 weighted number of women) of the reproductive age women had their last live birth in a health facility. This rate slightly increased to 6.4% (468/7307) in 2005 and increased by almost two-folds to 11.8% (933/7908) in 2011.

By the year 2000, the rate of birth in a health facility among urban women in Ethiopia was 33.4% compared with only 1.8% among their rural counterparts. Since 2000, the rates have been persistently increasing among both urban and rural women. Model 1, however, shows that the gap between the two groups had persisted, giving urban vs. rural OR range between 24.0 in 2011 and about 31 in 2005 [Table 1].

**Table 1:** Weighted rates per 100 of birth in a health facility by study predictors among women in Ethiopia.

Characteristics	Year								
	2000			2005			2011		
	Number	Rate	Crude OR (95% CI)	Number	Rate	Crude OR (95% CI)	Number	Rate	Crude OR (95% CI)
**Residence**	**7969**			**7273**			**7875**		
Urban	908	33.4	26.7(21.4,33.3)^c^	631	46.0	31.2(25.1,38.7)^c^	1186	53.0	24.0(20.4,28.2)^c^
Rural	7061	1.8	1	6642	2.7	1	6689	4.5	1
**Region**	**7970**			**7271**			**7876**		
Amhara	2223	3.5	1	1855	4.2	1	1990	11.0	1
Tigray	535	4.7	1.3(0.9,2.1)	480	7.7	1.9(1.3,2.8)^b^	527	13.9	1.3(1.0,1.7)
Oromiya	3060	4.0	1.1(0.8,1.5)	2709	5.1	1.2(0.9,1.6)	3101	9.3	0.8(0.7,1.0)
SNNPR	1690	4.1	1.1(0.8,1.6)	1624	4.2	1.0(0.7,1.4)	1630	7.9	0.7(0.6,0.9)^b^
Afar, Harari, Somali	186	8.1	2.4(1.3,4.2)^b^	370	7.3	1.8(1.1,2.8)^a^	295	10.8	1.0(0.7,1.5)
Ben-Gumuz, Gambela	102	12.7	3.8(2.0,7.1)^c^	81	9.9	2.5(1.1,5.3)^a^	115	16.5	1.6(1.0,2.7)
Addis Ababa, Dire Dawa	174	62.6	44.8(30.7,65.5)^c^	152	73.0	61.1(40.0,93.3)^c^	218	78.0	28.8(20.3,40.9)^c^
**Economic status**	**7641**			**7272**			**7876**		
Poorest	1705	1.1	1	1508	0.7	1	1730	2.5	1
Poorer	2064	1.2	1.1(0.6,1.9)	1545	1.4	1.9(0.9,3.9)	1694	3.4	1.3(0.9,2.0)
Middle	1063	2.1	1.9(1.0,3.5)	1585	1.9	2.7(1.3,5.4)^b^	1617	3.3	1.3(0.9,2.0)
Richer	1759	3.0	2.7(1.6,4.6)^c^	1445	4.6	6.6(3.4,12.5)^c^	1488	7.5	3.2(2.2,4.5)^c^
Richest	1050	26.6	32.1(20.0,51.5)^c^	1189	28.5	55.0(29.9,101.2)^c^	1347	49.1	37.5(27.2,51.6)^c^
**Education**	**7969**			**7271**			**7875**		
No education	6543	2.2	1	5708	2.6	1	5250	4.9	1
Primary	1003	9.9	4.8(3.7,6.3)^c^	1197	10.0	4.2(3.3,5.4)^c^	2260	17.9	4.3(3.6,5.0)^c^
Secondary and higher	423	44.4	35.1(27.3,45.2)^c^	366	54.4	45.0(34.6,58.5)^c^	365	73.2	53.6(41.2,69.8)^c^

a p-value <0.05,

b p-value <0.01,

c p-value <0.001.

Place of residence is moderately correlated with household economic status and is weakly correlated with education. Economic status and maternal education are weakly correlated with each other (data available on request). In Model 2, when household economic status and education of the mother are included as factors, the association between place of residence and birth in a health facility is weakened. However, the association remains strong. Moreover, all the standard errors for the coefficients are less than 2 suggesting absence of multicollinearity (data available on request).

**Figure 1:**
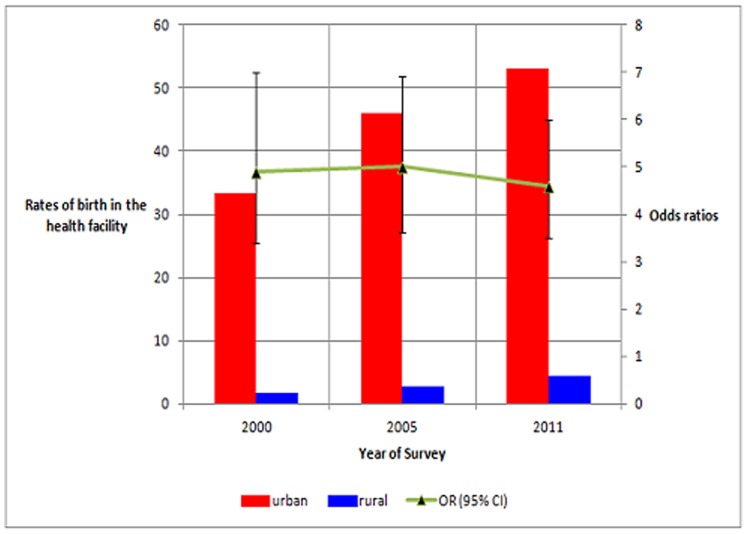
Birth in a health facility: inequalities between women of urban- and rural-areas of Ethiopia. n = 6842, 2000; n = 6389, 2005; and n = 7550, 2011. Adjusted for age, attitude towards spousal violence, economic autonomy, and antenatal care status.

Model 3 builds on model 2 and by controlling for age, economic autonomy, attitude towards spousal violence, and ANC status, it shows that ORs of birth in a health facility among urban relative to rural women were 4.9 (95% CI: 3.4, 7.0, p<0.0001) in 2000 and 4.6 (95% CI: 3.5, 6.0, p<0.0001) in 2011[Figure 1].

In line with the urban/rural disparities, the highest rates of birth in health facilities were noted in Addis Ababa and Dire Dawa, standing at 62.6% in 2000 and increasing to 78% in 2011. Rates were also observed to increase in all the other administrative regions; for example, the lowest-ranking rate of the Amhara administrative region tripled during the study period from 3.5% in 2000 to 11.0% in 2011. In the year 2011, the rate of Amhara administrative region was similar to all the other administrative regions (Tigray, Oromiya, SNNPR, Afar, Harari, Somali, Ben-Gumuz, and Gambela), with the exception of Addis Ababa and Dire Dawa [Table 1].

Administrative region is weakly associated with household economic status and maternal education (data available on request). In model 2, the crude effect of administrative region is slightly weakened when the effects of household economic status and maternal education are removed (data available on request).

In model 3, controlling of the effects of age and economic autonomy, the successive OR of birth in a health facility among women from Addis Ababa and Dire Dawa regions compared to Amhara region was 5.7 (95% CI: 3.5, 9.3, p<0.0001) in 2000 increasing to 13.6 (95% CI: 8.2, 22.6, p<0.0001) in 2005 before dwindling to 6.4 (95% CI: 4.3, 9.6, p<0.0001) in 2011 [Table 2].

**Table 2:** Adjusted ORs and 95% CIs of disparities in the use of health facility for birth by administrative region and household economic status among women in Ethiopia.

	Year					
	2000		2005		2011	
Characteristics	Adjusted OR (95% CI)	p-value	Adjusted OR (95% CI)	p-value	Adjusted OR (95% CI)	p-value
**Region** *						
Amhara	1		1		1	
Tigray	1.0 (0.6, 1.6)	0.967	1.5 (0.9, 2.3)	0.106	0.9 (0.6, 1.2)	0.369
Oromiya	0.7 (0.5,0.9)	0.021	1.0 (0.8, 1.4)	0.829	0.6 (0.5, 0.7)	<0.0001
SNNPR**	0.9 (0.6,1.3)	0.428	0.8 (0.6, 1.2)	0.343	0.5 (0.4, 0.6)	<0.0001
Afar, Harari, Somali	2.1 (1.04, 4.1)	0.031	3.2 (1.9, 5.5)	<0.001	0.9 (0.6, 1.5)	0.718
Benshangul-Gumuz, Gambela	2.9 (1.4, 5.8)	0.004	2.1 (0.9, 5.0)	0.083	1.6 (0.9, 2.9)	0.143
Addis Ababa, Dire Dawa	5.7 (3.5, 9.3)	<0.0001	13.6 (8.2, 22.6)	<0.0001	6.4 (4.3, 9.6)	<0.0001
**Economic status** ***						
Poorest	1		1		1	
Poorer	1.1 (0.6, 2.1)	0.673	1.7 (0.8, 3.1)	0.176	1.1 (0.7, 1.7)	0.648
Middle	1.3 (0.7, 2.4)	0.481	2.0 (1.0, 4.0)	0.056	1.1 (0.7, 1.6)	0.794
Richer	1.8 (1.1, 3.2)	0.027	4.1 (2.1, 7.9)	<0.0001	1.5 (1.0, 2.1)	0.050
Richest	3.0 (1.7, 5.4)	0.0001	7.2 (3.7, 14.1)	<0.0001	3.8 (2.5, 5.6)	<0.0001

*n = 6860, 2000; n = 6405, 2005; n = 7591, 2011 (adjusted for age and economic autonomy).

**SNNPR = Southern Nations, Nationalities, and Peoples Region.

***n = 6842, 2000; n = 6389, 2005; n = 7550, 2011 (adjusted for age, economic autonomy, attitude towards spousal violence, and ANC status).

Note: n is not weighted while the ORs are weighted.

During the study period, the rates of birth in a health facility increased across all household economic strata in Ethiopia; rates increased from 1.1% in 2000 to 2.5% in 2011 among women from the poorest households, and from 26.6% to 49.1% among women of the richest households. There was a monotonic increase in the percentage of women who gave birth in a health facility with an increase in household economic status [Table 1].

Removing the effects of place of residence and maternal education, and controlling for age, economic autonomy, attitude towards spousal violence, and ANC status, the independent OR for birth in a health facility comparing women of the richest households with those of the poorest households were 3.0 (95% CI: 1.7, 5.4, p = 0.0001), 7.2 (95% CI: 3.7, 14.1, p<0.0001), and 3.8 (95% CI: 2.5, 5.6, p<0.0001) in 2000, 2005, and 2011, respectively [Table 2]. In this model, however, there were no statistically significant differences in the use of health facility for birth between women from the poorest-, poorer-, and middle-economic status households.

**Figure 2:**
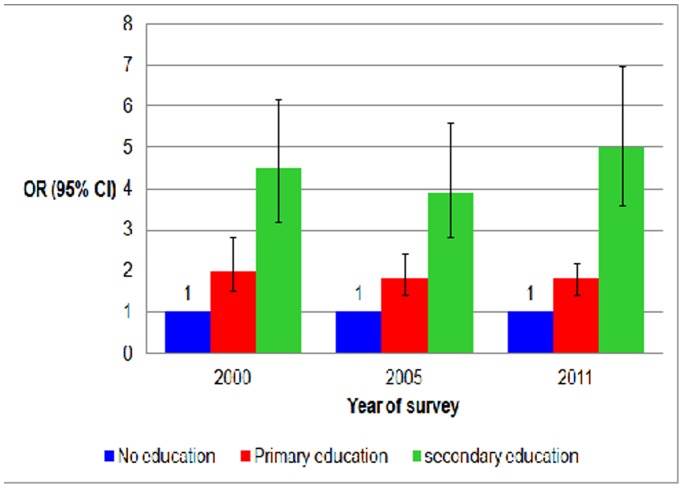
The use of health facility for birth –the education divides among women in Ethiopia. n = 6842, 2000; n = 6389, 2005; and n = 7550, 2011, AOR = Adjusted Odds Ratio, CI = Confidence Interval. Adjusted for age, attitude towards spousal violence, economic autonomy and antenatal care status.

Besides inequalities in health facility birth in Ethiopia based on area of residence, administrative region and household economic status, we have also observed differences based solely on the maternal education. During the study period, the level of illiteracy dropped from 82% to 66%, and rates of primary education have increased. Women of secondary or higher education have remained a negligible part of the population (about 5%). Nevertheless, the rate of birth in a health facility among women with a secondary or higher education has increased from about 44% in 2000 to around 73% in 2011. On the contrary, during the same time period, the change among women of no education was trivial, increasing from about 2% to nearly 5%. The OR of health facility birth of women with secondary or higher education relative to women of no education has shown a rising trend from 35.1(95% CI: 27.3, 45.2, p<0.05) in 2000 to 53.6(95% CI: 41.2, 69.8 p<0.05) in 2011 [Table 1]. Education has remained an independent and statistically significant predictor of birth in a health facility after removing the effects of area of residence and household economic status, and controlling for the potential confounding effects of age, economic autonomy, attitude towards spousal violence and ANC status with OR of 4.5 (95% CI: 3.2, 6.2, p<0.0001), 3.9 (95% CI: 2.8, 5.6, p<0.0001) and 5.0 (95% CI: 3.6,7.0, p<0.0001) among women with secondary/higher vs. no education in 2000, 2005, and 2011, respectively [Figure 2].

## Discussion

During a period of fifteen years, a substantial improvement in the overall rate of birth in a health facility in Ethiopia was demonstrated, nevertheless, the overall rate as of 2011, was considerably low, standing at 12%. An absolute improvement was observed in both urban and rural areas, as well as across all administrative regions, economic strata and levels of education. However, our study highlights the deprivation of women from rural areas; most administrative region except Addis Ababa and Dire Dawa; the poorest, poorer or middle economic status households; and no education who are doing the heavy lifting in Ethiopia by giving birth at home in unsafe conditions. The problem is deep rooted and has not changed significantly even with the increasing expansion of primary health care facilities in the country [3]. Moreover, the proliferation of the private sector is limited to urban areas [19]. However, the role of the private sector in the inequality equation is uncertain.

Moreover, gaps between urban and rural areas were sustained over the years because the increase in rural areas of the number of women giving birth in a health facility is trivial. In rural areas, still less than 5% of the mothers give birth in health facilities, highlighting a tremendous room for improvement. Our findings of urban/rural disparities were confirmed by a study from India where 67% of women from urban residential areas give birth in health institutions compared to only 29% of women living in rural areas [20]. Nevertheless, the urban rural divide in Ethiopia is wider than the one reported for India. The persisting urban-rural differences in Ethiopia are mediated by a larger and more pervasive problem which is the long distances between rural residences and health facilities. According to Bailey, 75% of the population of northern Ethiopian regions (Amhara and Tigray) live in areas which are more than 3 hours walking distance from the nearest primary health care units (facilities which do not provide cesarean section services) [21].

There was a sign of closing gaps between administrative regions, except for Oromia and SNNPR which were left behind during the course of the fifteen years. Even though the historical concentration of modern healthcare around city states (Addis Ababa and Dire Dawa) explains the gap between these states and Amhara, why SNNPR and Oromia administrative regions are underperforming in expanding care compared to Amhara is unclear.

After an increase between 2000 and 2005, the disparity between women of the poorest and the richest households is on decline. It was also noted that, women from the poorer and middle economic status households have no advantage compared to the poorest women, highlighting the absence of advantage for the middle class. A study from Morocco has shown that higher economic status is positively associated with delivery in a medical setting, with women from above the median economic status households being 3 times more likely to give birth in a health facility compared with women from the households whose wealth status was below the median [22]. Similar findings were reported from Kenya, where women from the richest households having about 6 times higher odds of birth in the health institution than their counterparts from the poorest households [23]. Furthermore, a study in Nepal, which has measured economic status using household assets, such as type of latrine, cooking fuel, ownership of television and telephone, has corroborated our findings, showing that women from low economic status households to be 4.4 times more likely to give birth at home relative to women from high economic status households [24]. Payment for maternity services by poor women might be feeding into the economic disparities. Even though delivery care in health facilities is supposed to be free in Ethiopia, the majority of the health centers in this country charge clients for some aspect of care. So could transportation to reach a health facility is paid by the women. Pearson and colleagues surveyed 751 health facilities that have provided child birth services since 2008 in Ethiopia. It was reported that 68% of the facilities have charged in cash or kind for normal delivery [25].

Systematic disparities between women of secondary or higher education and no education were sustained over the years. On the contrary, difference between women of primary education and no education is narrowing. It was demonstrated now and then in different countries and settings that there is an inequitable birth in a health facility on the basis of educational status of women. For example, in southern Tanzania, in 2004, mothers with primary or higher education were 1.29 (95% CI: 1.20–1.38) times more likely to give birth in health facility than mothers with no education, adjusting (among other factors) for age, gender of head of the household, ethnic group and household economic status [26]. In the Matlab province of Bangladesh, in 2002, the OR for facility-based delivery for women of secondary or higher education compared to no education was 2.69 (95% CI: 2.26–3.20) after adjusting for, among other factors, age, gravidity, distance and economic status [27]. Nonetheless, the economic and educational disparities are wider in Ethiopia than those found by the studies from Nepal, Tanzania, and Bangladesh. Women with no education are lagging; this might be mediated by health knowledge that increasing levels of education are associated with increasing health knowledge. Greenaway noted that, in Ghana, health knowledge as measured by “correctly identifying four myths about Human Immunodeficiency Virus, three misunderstandings about the transmission of Tuberculosis, and listing up to 7 forms of modern contraception” explains a portion of the link between formal education and use of health services (ANC, giving birth attended by skilled provider, complete child vaccination combined) [28].

The findings of this study are limited by the fact that the study’s design was cross-sectional which made it difficult to establish temporality between the predictor variables and place of birth. This study is also constrained for its inability to control the effect the quality of child birth services has on the uptake. Nevertheless, the strengths of this study include the ability to generalize the findings to the national level and controlling of a handful of confounders that could affect the choice of place of birth including age, autonomy and ANC visits.

## Conclusions

Our study has demonstrated low rates of deliveries in health facilities with huge gaps in the choice of place of birth between urban and rural areas. Disparities between women of Amhara and other regions have slightly weakened, though disparities between Amhara and Addis Ababa/Dire Dawa seem to hold. The gap between women of the richer and the poorest households has initially strengthened to be weakened afterwards. Similarly, differences between women from richest households and from poorest households soared initially only to decline afterwards. But, the inequity between these groups at the end point, 2011, is marginally stronger than at the starting point, 2000. Furthermore, gap between women of secondary/above education and no education in terms of giving birth in a health facility is large and has been sustained over time. The federal government and administrative regions should put more effort to increase the use of healthcare facilities for deliveries by all women and reduce disparities by place of residence, economic- and educational-strata of the Ethiopian women.

Moreover, the federal government should do more to reduce the gaps between Addis Ababa/Dire Dawa and other administrative region.

Future studies are needed to examine why there is a stubborn gap between City states (Addis Ababa and Dire Dawa) and other regions in terms of giving birth in a health facility and for the absence of the difference between women of poorest- and middle-economic status households.
